# Prion Diseases: A Unique Transmissible Agent or a Model for Neurodegenerative Diseases?

**DOI:** 10.3390/biom11020207

**Published:** 2021-02-02

**Authors:** Diane L. Ritchie, Marcelo A. Barria

**Affiliations:** National CJD Research & Surveillance Unit, Centre for Clinical Brain Sciences, University of Edinburgh, Western General Hospital, Crewe Road, Edinburgh EH4 2XU, UK; marcelo.barria@ed.ac.uk

**Keywords:** neurodegenerative diseases, prion disease, transmission, amyloid-beta, protein misfolding, prion-like, iatrogenic, Alzheimer’s disease

## Abstract

The accumulation and propagation in the brain of misfolded proteins is a pathological hallmark shared by many neurodegenerative diseases such as Alzheimer’s disease (Aβ and tau), Parkinson’s disease (α-synuclein), and prion disease (prion protein). Currently, there is no epidemiological evidence to suggest that neurodegenerative disorders are infectious, apart from prion diseases. However, there is an increasing body of evidence from experimental models to suggest that other pathogenic proteins such as Aβ and tau can propagate in vivo and in vitro in a prion-like mechanism, inducing the formation of misfolded protein aggregates such as amyloid plaques and neurofibrillary tangles. Such similarities have raised concerns that misfolded proteins, other than the prion protein, could potentially transmit from person-to-person as rare events after lengthy incubation periods. Such concerns have been heightened following a number of recent reports of the possible inadvertent transmission of Aβ pathology via medical and surgical procedures. This review will provide a historical perspective on the unique transmissible nature of prion diseases, examining their impact on public health and the ongoing concerns raised by this rare group of disorders. Additionally, this review will provide an insight into current evidence supporting the potential transmissibility of other pathogenic proteins associated with more common neurodegenerative disorders and the potential implications for public health.

## 1. Introduction

Globally, average life expectancy has shown a sustained increase over the last four decades. Associated with this increase is a substantial rise in the prevalence of age-related disorders [1]. Ageing is a well-recognised risk factor for neurodegenerative diseases, a group of incurable, debilitating, and invariably fatal diseases that encompass a range of disorders, including Alzheimer’s disease (AD) and other dementias, Parkinson’s disease (PD), Huntington’s disease, Motor Neuron Disease (MND), and prion disease [2]. With the World Health Organisation (WHO) predicting that dementia alone will affect 135 million people by the year 2050, it is expected that the current global social and economic burden presented by this group of disorders will be exacerbated in future decades [3].

**Figure 1:**
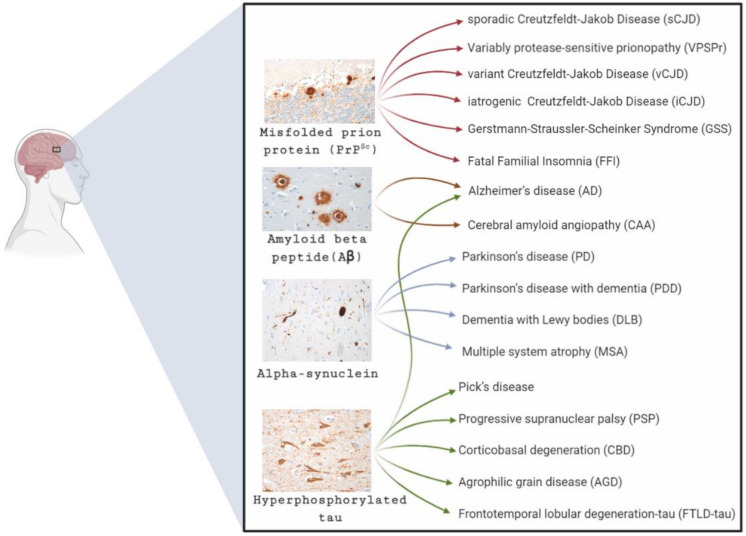
Common neurodegenerative diseases and the predominant protein accumulation in the brain. The most common misfolded proteins associated with neurodegenerative diseases are amyloid-beta (Aβ), tau, and alpha-synuclein (α-synuclein). These protein aggregates are represented in the four different micrographs. In some instances, the neurodegenerative conditions are associated with the accumulation in the brain of a single major protein type; this is best described in prion diseases. However, in other disorders such as Alzheimer’s disease, multiple major protein forms are described. With a number of conditions characterised by the same protein aggregation, differential diagnosis is often dependent on the clinical phenotype.

The vast majority of neurodegenerative diseases are associated with the intracellular or extracellular accumulation of misfolded protein within the brain [4]. These abnormal protein deposits lead to the dysfunction and subsequent loss of the neuronal population, resulting in the progression of a wide range of clinical symptoms that may include cognitive decline, dementia, and a gradual loss of locomotor functions. Whilst different neurodegenerative conditions are associated with disease-specific proteins such as amyloid-beta (Aβ) and hyperphosphorylated tau (tau) in AD, α-synuclein in PD, and TAR DNA-Binding Protein 43 (TDP-43) in MND, a common molecular mechanism is proposed to underlie the replication and spread of these different misfolded protein aggregates in the central nervous system (CNS) (Figure 1) [5,6]. The archetypal model for this mechanism is that described in prion diseases, a rare group of neurodegenerative diseases that occur in humans as well as a number of animal species. In prion diseases, a misfolded and abnormal form of a normal endogenous protein, the prion protein (PrP^C^), interact to form β-sheet rich structures with the propensity to aggregate in the CNS. The misfolded prion protein, termed prion (PrP^Sc^), forms a seed or template that converts further PrP^C^ monomers into the abnormal and disease-associated isoform [5,6].

At present, there is an increasing body of evidence illustrating a commonality between the properties of pathogenic proteins such as Aβ, tau, and α-synuclein with those demonstrated by prions. As a consequence, neurodegenerative diseases including AD and PD, are increasingly referred to as “prion-like” disorders [7]. The precise definition of “prion” is “proteinaceous infectious particle”, in recognition of the unique infectious nature of prions, in which they have shown to transmit not only between species but in some instances across different species [8]. With no large-scale epidemiological studies suggesting that other neurodegenerative disorders are infectious, apart from prion disease, the categorisation of disorders such as AD and PD as prion-like remains somewhat contentious. However, advocates of this categorisation point to an increasing body of evidence from experimental models demonstrating that protein aggregates such as Aβ and tau can propagate in vivo and in vitro in a prion-like mechanism generating misfolded protein aggregates such as amyloid plaques and neurofibrillary tangles. Furthermore, over the last five years, a number of case reports have documented the possible inadvertent human transmission of Aβ via medical and surgical procedures, heightening concerns that misfolded proteins, other than the prion protein, could potentially transmit from person-to-person as rare events and with prolonged incubation periods [9].

In this review, we will provide a historical perspective on the unique transmissible nature of prion diseases, examining their impact on public health and the ongoing concerns raised by this rare group of disorders. Additionally, this review will provide an insight into current evidence supporting the potential transmissibility of other pathogenic proteins associated with more common neurodegenerative disorders and the possible implications for public health.

## 2. Prion Diseases

Prion diseases are a rare but unique group of neurodegenerative disorders that occur naturally in humans as well as a number of animal species. Creutzfeldt-Jakob disease (CJD) in humans, scrapie in sheep and goats, bovine spongiform encephalopathy (BSE) in cattle and chronic wasting disease (CWD) in cervids are just a few of the more familiar forms of prion disease. Human and animal prion diseases are characterised by a common pathology that includes spongiform degeneration in cerebral grey matter regions, reactive proliferation of glial populations, neuronal loss and the accumulation in the CNS of a misfolded and disease-associated form of the prion protein, termed PrP^Sc^ [10]. Human prion diseases, like other neurodegenerative conditions occur largely as sporadic or genetic conditions: indeed, sporadic Creutzfeldt-Jakob disease (sCJD) is the most common form, accounting for approximately 85% of all human prion diseases [11]. In contrast, a small proportion of human prion diseases (<1%) are acquired and include kuru, iatrogenic CJD (iCJD) and variant CJD (vCJD). This differs to that of naturally occurring animal prion diseases in which the majority of forms are acquired. With animal prion diseases also spreading as zoonotic infection to humans, as identified with the emergence of vCJD [12], enormous public and scientific attention has focused on this rare group of diseases, not only over concerns for the possible human-to-human transmission but also the potential risks to human health raised by animal diseases.

### 2.1. Prion Diseases, a Brief Historical Perspective

Prion diseases have a long-recorded history, dating back to the 18th century with the first descriptions of scrapie, a naturally occurring neurological disease of sheep and goats [13]. More than two centuries later, scrapie remains the archetypal prion disease, with the misfolded and disease-associated form of the prion protein still referred to as the scrapie isoform (PrP^Sc^) by a large proportion of the scientific community. Seminal in vivo and in vitro investigations led by Stanley Prusiner using scrapie-infected tissue were instrumental in characterizing many of the properties associated with prion diseases, including (i) the transmissibility between individuals of the same species and in some instances across species [14,15,16], (ii) the identification that a protein forms the major, if not sole, component of the infectious agent [8,17,18,19], demonstrating a resistance to proteolytic digestion [8,17,18,19], high temperatures [20], and conventional decontamination methods [21,22,23], (iii) the propensity of the misfolded and disease-associated prion protein to aggregate in the CNS, (iv) the prolonged asymptomatic incubation periods associated with the disease [14,15], (v) the existence of multiple isolates or strains of prions with diverse biological properties [24,25,26] and (vi) the phenomenon of the “species barrier”, more commonly referred to as transmission barrier, in which there may be inefficient transmission of infectivity between different species [27].

Early studies of scrapie also provided the first clues to the aetiology of human prion diseases with the observations by William Hadlow and colleagues that the neuropathological features described in scrapie were strikingly similar to that of kuru, an obscure progressive neurological disease that was endemic amongst the Fore-linguistic tribes of the Eastern highlands of Papua New Guinea during the 1950s [28]. With the transmissibility of scrapie already established, Hadlow raised the possibility that kuru may also be a transmissible disease. This transmissibility was confirmed several years later with a seminal study by Carleton Gajdusek, Clarence Gibbs, and Michael Alpers, in which they described the development of a kuru-like illness in non-human primates experimentally inoculated with brain isolates of a kuru case, confirming for the first time that a human neurological disease could be transmissible [29]. Gajdusek and colleagues extended the evidence for human transmissible prion diseases with the subsequent intracerebral injection of non-human primates with brain homogenates from CJD patients, and later with brain isolates from patients with Gerstmann–Sträussler–Scheinker (GSS) syndrome [30,31].

### 2.2. Models of Prion Disease

#### 2.2.1. Animal Models

Experimental animal models have become a mainstay in prion disease research and have been employed to address many aspects of prion disease [32]. The earliest experimental animal models used sheep, goats, and non-human primates and have resulted in a number of seminal studies including, the first evidence of the transmissibility of animal and human prion diseases [14,15,29], the first recognition that BSE and vCJD shared a similar pathological phenotype [33] and more recently, experimental transmissions in sheep provided a valuable model in which to assess potential risks associated with blood transfusion [34,35,36]. 

The development of rodent models during the 1960s marked a milestone in prion disease research, providing a more economical and practical model in which to study prion disease with significantly shorter incubation periods compared to large animal models [37,38,39]. Initial studies using wild-type hamsters and mice were invaluable in providing an understanding of the biology of prion strains [40,41] and transmission barriers, as well an insight into the pathogenesis of the infectious agent having established an early involvement of the peripheral nervous system [42,43,44]. The strain phenomenon is one of the most intriguing features of prion disease and is best demonstrated following the intracerebral inoculation of wild-type mice with different scrapie isolates. Distinct strains of the transmissible agent were identified based on the individual biological properties of the inoculated mice (primarily by the incubation period and neuropathological phenotype), which are then stable on subsequent serial passage [40,41,45]. These strain properties have been accounted for by differences in the conformation of PrP^Sc^ but are also heavily influenced by the prion protein genotype, and in the case of human prion diseases in particular, the polymorphism at codon 129 on the human prion protein gene (*PRNP*). In a seminal study by MBruce and colleagues, the experimental transmission of vCJD brain isolates in wild-type mice resulted in transmission properties (incubation period and lesion profile) that were indistinguishable from those obtained with BSE but that differed from those obtained with sCJD [46]. This provided the first convincing evidence that these two diseases were caused by the same prion strain. Further studies on wild-type mice demonstrated that vCJD peripheral tissues (spleen and tonsil) known to contain demonstrable quantities of PrP^Sc^ were infectious to wild-type mice [47]. Over the last decade, wild-type mice continued to play an invaluable role in the strain characterisation of vCJD in cases identified in the UK and worldwide [48,49,50,51].

During the early 1980s, the generation of mice expressing novel transgenes ushered a new era, not only in prion research but in the understanding of other disease mechanisms, including other protein misfolding neurodegenerative disorders [52]. A large proportion of transgenic mice used in prion research have been developed to investigate and circumvent the species barrier with the development of mouse lines that expressed prion gene sequences from other species. In a seminal study by Prusiner and colleagues, transgenic mice expressing hamster PrP demonstrated significantly shorter incubation periods compared to wild-type mice when inoculated with a scrapie-infected hamster isolate [53]. While the mechanism of the species barrier is still not clear, it is thought to be heavily influenced by the degree of homology in the primary PrP amino acid sequence between the PrP^Sc^ of the donor and PrP^C^ of the recipient species, the physiological differences between the species in question, and the animal prion strain, as enciphered in the conformation of PrP^Sc^. In the investigation of human prion disease, different sCJD types were found to transmit inefficiently to wild-type mice, often without clinical symptoms [46]. The generation of different transgenic mouse lines expressing the *PRNP* gene provided the first opportunity to observe more extensive transmission characteristics of sCJD and other human prion diseases. There are two main methods for the generation of transgenic mice; random genetic insertion and gene targeting. In this regard, differences in the susceptibility to prion disease from transgenic mouse models have been observed. These differences can be partially attributed to the over-expression of the prion protein and to the insertion point of the prion protein gene into the recipient species. The gene targeting technology consist of the direct replacement of the mouse prion protein gene (*Prn-p*) gene with the genetic material for insertion. In the case of human transgenic mice, insertion of *PRNP*. A series of transmission experiments by the groups of Jean Manson and Tetsuyuki Kitamoto have used gene targeting in the development of several “humanised” transgenic mice, in which the *Prn-p* gene was directly replaced by *PRNP*. These mouse lines have been used to investigate specific familial mutations associated with disease as well as extensive studies on the effect of the naturally occurring methionine (M) to valine (M) polymorphism at codon 129 on *PRNP*, which is a recognised risk factor for human prion diseases. Overall, these two transgenic procedures have been invaluable in investigating human prion diseases and have enabled the study of the effects of the codon 129 polymorphism, assessing the risks for potential human-to-human transmission of prion disease, and evaluating the zoonotic potential from animal prion diseases [32].

#### 2.2.2. Cell-Free Conversion Assays

Over the past two decades, the cell-free conversion assays have been developed in a concerted effort to pursue alternatives to the use of animal models in prion research [54]. The introduction of these cell-free assays offered the potential for a more rapid and economical model in which to study a number of aspects relating to prion disease, combined with the ability to manipulate experimental conditions within a controlled environment. In basic terms, these methodologies allow the in vitro amplification of PrP^Sc^ present at low levels in tissues and biological fluids to levels that are detectable by conventional laboratory techniques.

Following several years of refinement, two cell-free assays are most widely used in prion disease.These are the protein misfolding cyclic amplification (PMCA) assay and Real Time Quaking Induced Conversion (RT-QuIC) assay. Fundamentally, these assays require a number of basic elements including, (i) a source of PrP^Sc^ (seed), (ii) an excess of PrP^C^ (substrate), (iii) a mechanism for energy input and, (iv) a suitable method in which to detect the amplified misfolded prion protein product. The PMCA assay was first described by Claudio Soto and colleagues in 2001 with the serial dilution of scrapie-infected brain homogenate (seed) into crude brain homogenate from uninfected Syrian hamsters (substrate) [55]. Following successive rounds of sonication (ultrasound) and incubation, amplified PrP^Sc^ was detected by conventional Western blot protocols following proteolytic treatment to detect the partially protease-resistant form of the prion agent, the PrP^res^ [55]. A number of modified PMCA protocols and aggregation assays have been developed and have proved a valuable tool in providing support of the protein-only hypothesis [56,57,58,59,60]. More recently, PMCA has proved a useful tool in the understanding of many aspects of prion biology; including, the effects of *PRNP* codon 129 genotype [61]; the species barrier and zoonotic potential of prions [62,63,64], and the propagation of prion strains [65,66]. PMCA has also shown great potential in the development of a diagnostic tool in the diagnosis of vCJD, having shown sufficient sensitivity and specificity to detect PrP^Sc^ in urine [67], blood [68,69,70], and recently in CSF samples [71,72] from vCJD cases. The RT-QuIC assay employs shaking rather that sonication during the amplification process. In a further variance to PMCA, RT-QuIC uses purified recombinant PrP as the substrate rather than brain homogenate or cell extracts used in PMCA, with the conversion and amplification of the recombinant PrP monitored in real time using the fluorescent dye thioflavin-T [73,74]. Due to the high sensitivity and specificity of RT-QuIC in the analysis of CSF samples from sCJD patients, RT-QuIC analysis is included in the clinical diagnosis of sCJD in the United Kingdom (UK) (https://www.cjd.ed.ac.uk/sites/default/files/criteria.pdf;) [75,76].

The development, optimisation, and multiple application of the cell-free assays have provided valuable information on the protein misfolding phenomena associated with protein misfolding disorders, principally involving animal and human prion diseases [54,77]. The complementation of these molecular assays along with experimental animal models will further continue providing evidence on the complexity surrounding the prion biology and a further understanding of other neurodegenerative disorders related to protein misfolding.

### 2.3. Animal Prion Diseases and Their Zoonotic Potential

Since the first descriptions of scrapie in sheep, a range of animal prion diseases have been identified including BSE in cattle, CWD in cervids, transmissible mink encephalopathy (TME) in farmed mink, and feline spongiform encephalopathy (FSE) in domestic and exotic cats. In contrast to human prion diseases, the vast majority of animal prion diseases have an infectious aetiology. This has inevitably raised concerns over the potential cross-species transmission to humans, particularly through possible dietary exposure to the infectious agent [78].

To date, there is no robust epidemiological evidence to suggest that naturally occurring prion disease of sheep are a risk factor in the development of human prion disease. This is largely based on early prevalence studies that have reported the incidence of CJD in countries considered scrapie-free (Australia and New Zealand) to be similar to countries where scrapie remains endemic in sheep [79]. Likewise, inoculation of scrapie isolates in non-human primates and in humanised transgenic mice expressing wild-type levels of PrP have provided limited evidence of transmission, consistent with the presence of a significant species barrier protecting humans from infection with scrapie prions [80,81,82,83,84]. However, the recent demonstration of PrP^Sc^ within the brain of a proportion of scrapie challenged transgenic mice overexpressing human PrP, and the development of a clinical disease in a non-human primate intracerebrally inoculated with natural scrapie isolates, suggest that the zoonotic potential of scrapie prions to humans cannot be discounted [85,86].

In the wake of the BSE epidemic in the UK during the 1980s, concerns over the cross-species transmission of BSE to humans resulted in the UK government instigating a national surveillance program to monitor any changes in the incidence or phenotype of human prion diseases. In 1996, through this national program, a novel human prion disease, referred to as vCJD, was described in patients of an uncharacteristically young age with a much longer disease duration than that typically observed in sCJD [12]. The emergence of this novel prion disease so closely following the BSE epidemic inevitably raised concerns that vCJD may have resulted from human exposure to the BSE agent through the consumption of contaminated meat products. This hypothesis was confirmed following experimental transmission in wild-type and transgenic mouse models, and in non-human primates, which demonstrated that BSE and vCJD had indistinguishable transmission properties, indicating a single strain of agent associated with these two prion diseases [33,46,87,88,89]. Currently, 232 clinical cases of vCJD have been identified worldwide, the majority of which have been reported in the UK (n = 178) [http://www.cjd.ed.ac.uk/sites/default/files/worldfigs.pdf]. Of the 232 vCJD cases with genetic analysis, all but a single case has occurred in individuals homozygous for methionine at codon 129 on *PRNP*. The remaining vCJD case, and the most recently identified in the UK, marked the first clinical case of pathologically confirmed vCJD to be recognised in a methionine/valine heterozygote (MV) individual [90]. The subsequent transmission of brain tissue from this vCJD MV individual to wild-type and transgenic mice has supported earlier experimental evidence in the same mouse model, demonstrating that other *PRNP* codon 129 genotypes are susceptible to infection with the BSE agent but may be subject to prolonged incubation periods [50,91,92]. Since the introduction of several strict control measures in the UK during the 1980s, only rare cases of BSE have been reported over the last few decades.

Current concerns over the zoonotic potential of animal prion diseases have focused on CWD, a highly contagious prion disease of cervids. CWD was first described in 1967 as an unusual wasting disorder affecting mule deer in Colorado [93]. Since this original description, CWD has been reported in a wide range of wild and farmed cervid species, spreading across 26 North American states and three Canadian provinces [94]. CWD is the most contagious animal prion diseases, spreading directly through animal-to-animal contact and indirectly through environmental contamination, with infectivity detected in bodily fluids and excreta including, placenta, saliva, faeces, and urine [95,96,97]. Of particular concern when assessing the zoonotic potential of CWD is the presence of PrP^Sc^ in peripheral tissues, many of which are included in the human food chain such as skeletal muscle, heart, and kidney [98]. Furthermore, experimental transmission studies have demonstrated the presence of multiple CWD strains, increasing the uncertainty over the potential cross-species transmission [99]. However, while experimentally, CWD has been transmitted to a range of animal species including ferret, racoon, mink and cattle, the risk of CWD transmission to humans is thought to be low [100]. Currently, epidemiological studies have provided no evidence of an association between CWD and the prevalence of human prion disease, and no novel human prion diseases have been identified in any North American state where CWD is endemic [101,102,103,104]. Likewise, experimental inoculation of CWD-infected brain isolates to transgenic mice expressing human PrP have failed to show substantial evidence of transmission, while transmissions to non-human primates have provided variable results [97]. Experimental modelling of the zoonotic potential of CWD using cell-free assays, particularly the PMCA assay reported molecular compatibility of CWD PrP^Sc^ using human PrP^C^ as a substrate [63,105,106,107,108].

While these studies are reassuring and suggest a significant species barrier between humans and CWD in cervids, the recent identification of CWD in free-ranging reindeer, moose, and red deer in Europe (Norway and Finland) has heightened concerns over the zoonotic potential, particularly as early indications suggest that the European cases of CWD may represent a new strain of CWD prions [109,110,111]. The extent of these concerns can be demonstrated by the implementation of a culling program in the affected reindeer population, introduced as an attempt to contain this outbreak. Experimental analysis and transmission studies involving infected material from the European cases are ongoing in several laboratories, results of which will provide a much clearer picture of the relationship between CWD cases found in Europe with those found in North America and the zoonotic potential of these recently identified cases.

### 2.4. Acquired Human Prion Diseases

**Figure 2:**
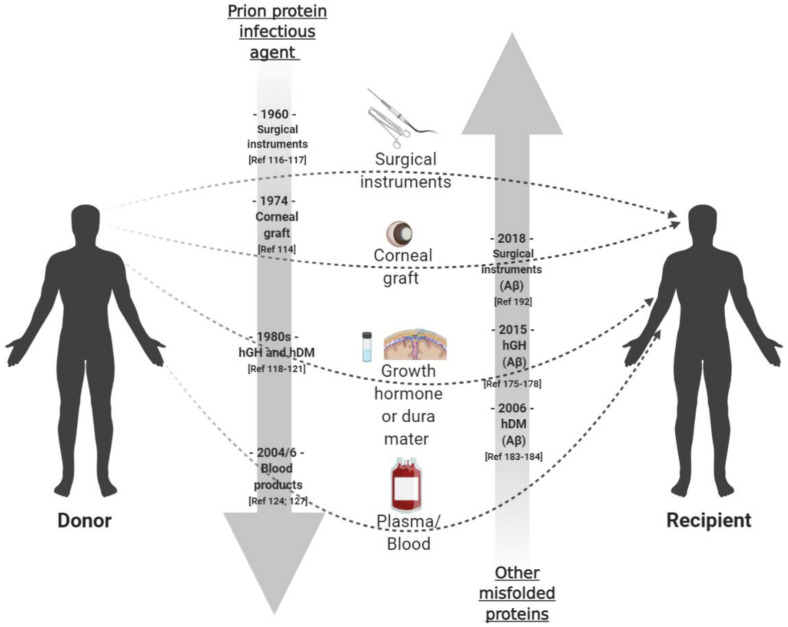
Schematic timeline (grey arrows) of the first reporting (as referenced) of acquired forms of Creutzfeldt-Jakob disease (CJD) and the possible human-to-human transmission of amyloid-beta (Aβ) pathology. The routes of exposure to prion protein aggregates, resulting in iatrogenic CJD (iCJD), are well-documented and include medical interventions such as cadaveric human growth hormone (hGH), cadaveric dura mater grafting (hGH), and through a small number of neurosurgical procedures. The recent re-examination of brain tissue from iCJD cases have suggested that Aβ neuropathology may also spread through similar routes of exposure. Concerns that the Aβ neuropathology may be a consequence of cross-seeding with co-existing prion protein aggregates were discounted following the observation of Aβ neuropathology in a proportion of hGH recipients who died from causes unrelated to CJD. While secondary transmission of variant CJD (vCJD) via transfusion medicine has been reported, no such instances of transmission have been identified for other pathogenic proteins. However, as the timeline for the identification of possible iatrogenic transmission of Aβ neuropathology occurs decades after that of acquired forms of CJD, the possibility of the transmission Aβ neuropathology via transfusion medicine requires further investigation, particularly due to the high presence of Aβ that accumulates in cerebral blood vessels in the iCJD patients.

While human prion diseases are rare, their transmissibility, lengthy incubation periods, and resistance to conventional decontamination methods present a considerable risk to public health. Kuru was the first human prion disease shown to be transmissible between humans [29]. However, the potential public health risks from the spread of kuru to the wider population were limited by the mode of transmission; specifically, by the ritualistic consumption of the brain and other tissues of the deceased during funeral ceremonies [112]. Following an imposed cessation on the practice of endocannibilism in the mid-1950s, numbers of kuru cases started to decline, and the disease is now considered extinct. The final deaths from kuru were recorded in 2003, over 50 years after the practice of endocannibilism was prohibited, providing a startling example of the lengthy incubation periods associated with this group of disorders [113]. Of greater concern to the wider population came in 1974 with a case report describing the first transmission of CJD in a patient who had received a corneal graft from a donor who had died from sCJD [114]. Retrospective review of case notes and subsequent reports described a small number of additional cases of iCJD in a further recipient of corneal graft and in patients operated on with contaminated neurosurgical instruments or stereotactic electroencephalogram electrodes (Figure 2) [115,116,117]. However, the greatest numbers of iCJD cases recorded worldwide (> 400 cases) have occurred in recipients of contaminated cadaveric human growth hormone (hGH) or human gonadotrophin hormones, and in recipient of human dura mater (hDM) grafts [118] (Figure 2). Following the identification of the first hGH-associated iCJD cases in the mid-1980s [119,120,121], the administration of hGH in the UK, and all other pituitary hormones was immediately stopped and replaced with recombinant pituitary hormones. Similarly, the use of commercially distributed cadaveric dura mater was replaced following the first recognition of hDM-iCJD in 1987. Despite these mitigations, cases of hGH-iCJD still occur in the UK, with the most recent death reported in 2020, reminiscent of the 40 years or more incubation periods that were first observed in kuru [122,123].

More recently, secondary transmission of vCJD was established with three clinical cases of vCJD associated with blood transfusion [124] (Figure 2). All three cases were identified through the Transfusion Medicine Epidemiology Review (TMER) project, a look back study established in the UK in 1997 in response to the emergence of vCJD, to look for any evidence that CJD may have transmitted via the UK blood supply. While epidemiological studies had provided no evidence that sCJD was transmissible via blood transfusion [125,126], the detection of infectivity in vCJD lymphoreticular tissues [47] had demonstrated that vCJD had a very different pathogenesis to sCJD, and one that could make vCJD more susceptible to this route of transmission. This was further supported by animal studies demonstrating that BSE could be experimentally transmitted via transfusion with blood collected from infected animals during the clinical and asymptomatic phase of the disease [36]. The three cases of secondary vCJD identified by the TMER study occurred in patients who had received transfusions with non-leukodepleted red cell concentrates from asymptomatic donors who subsequently developed vCJD [124]. Retrospective analysis of medical records from the blood donors and recipients showed an incubation period of approximately 7–9 years in the blood recipients and the period prior to clinical signs in the donors of 1–3 years. 

In 2004, a case of asymptomatic vCJD infection was reported in the UK in a patient who died from a non-neurological disorder five years after receiving a red cell transfusion from a donor who subsequently developed vCJD [127] (Figure 2). While no evidence of PrP^Sc^ was detected in the brain of this individual, biochemical and immunohistochemical analysis showed PrP^Sc^ accumulation in the spleen and cervical lymph node [127]. Subsequent inoculation of spleen tissue in wild-type and transgenic mice expressing human PrP confirmed infectivity associated with the spleen in this case [128]. A significant feature of this case was the MV heterozygosity at *PRNP* codon 129. With all clinical vCJD cases reported previously having been methionine homozygote at codon 129 (MM), this suggested that MV individuals may be susceptible to vCJD infection but with extended incubation periods. Following this report, a second suspected case of asymptomatic vCJD in a *PRNP* codon 129 MV individual was reported in a haemophiliac patient, which raised additional concerns over the safety of UK plasma products [129]. In 2016, the first clinical case of pathologically confirmed vCJD in a *PRNP* codon 129 individual was reported confirming that other *PRNP* genotypes were susceptible to vCJD infection [90]. This was supported by experimental transmission in transgenic mice, which demonstrated that susceptibility to vCJD varies according to the host genotype [50,92].

### 2.5. Current Public Health Risks From Human Prion Diseases

Clinical cases of vCJD have been in decline, having reached a peak in 2000 with 28 deaths. While this may be reassuring, the identification of vCJD in a *PRNP* codon 129 heterozygous individual, the last reported case of vCJD in the UK, raised concerns over a potential second wave of vCJD in this genotype [90]. Additionally, the detection of infectivity in lymphoreticular tissues during the long asymptomatic incubation periods [47,128] and reports of transfusion-transmission of vCJD infectivity from asymptomatic vCJD patients [124], questioned the numbers of the UK population harboring asymptomatic vCJD infection and the possible risk of further secondary human transmission via blood transfusion and potentially from surgery. In the absence of a blood-based assay for vCJD, three retrospective studies investigating PrP accumulation in formalin-fixed appendix tissue were undertaken to address these concerns [130,131,132,133]. This followed the observation of PrP positivity in appendix tissue removed from two patients that went on to develop clinical vCJD eight months and two years after their appendectomies [134]. Positive staining for the prion protein was reported in appendix specimens examined in all three studies and has provided a current estimated prevalence of asymptomatic vCJD infection in the UK of approximately 1 in 2000 of the population [131,133]. Genetic analysis of the positive appendix samples showed PrP accumulation in appendix specimens from all possible *PRNP* codon 129 genotypes [133,135], thus supporting data from experimental animal studies that showed all three *PRNP* codon 129 genotypes are susceptible to vCJD infection but may be subject to lengthy incubation periods [92]. Interestingly, data from the most recent appendix study showed PrP positivity in appendix specimens collected prior to the BSE epidemic and in specimens collected from patients born following the implementation of measures aimed at protecting the human food chain [132,133]. The identification of these positive specimens hypothesised that dietary BSE exposure in the UK population may have occurred over a wider time period that initially thought or that there may be a low prevalence of abnormal PrP in lymphoreticular tissues that does not progress to vCJD [133]. There remains some uncertainty over the interpretation of the abnormal PrP detected in the appendix in this series of prevalence studies in relation to vCJD infectivity. However, the detection of vCJD PrP^Sc^ in tissues and organs remains a surrogate marker of infectivity, which is largely supported by bioassay in experimental animal models [47,128,136]. Therefore, the prevalence of asymptomatic vCJD in the UK population estimated as high as 1 in 2000 and potentially over a larger proportion of the population continues to have a significant impact on public health concerns and emphasises the continued importance of ongoing surveillance of human prion diseases in the UK.

## 3. Human-to-Human Transmission of Other Pathogenic Proteins

Extensive study of prion disease has demonstrated an ever-increasing number of properties shared with more common neurodegenerative conditions [137]. Notable amongst these are, (i) increasing age as a risk factor in the development of the disease, (ii) the presence of inherited and sporadic forms, (iii) a pathological hallmark characterised by the accumulation in the brain of misfolded and disease-associated protein, (iv) a common model of self-propagation and disease progression, in which abnormally folded isoforms of a disease-associated protein interacts to form β sheet structures that aggregate to form a seed that results in the formation of other abnormally folded isoforms, (v) the cell-to-cell spread of the aggregated protein in a systematic process along well-defined neuroanatomical pathways and (vi) the identification of different strains or variants of the misfolded proteins, suggesting the ability to aggregate in different conformational states [137]. Additionally, experimental evidence suggesting resistance to conventional decontamination protocols, a defining property of PrP^Sc^, has also been reported with Aβ from AD cases and α-synuclein in patients with multiple system atrophy (MSA) [138,139,140]. While more recently, the resistance of misfolded proteins to protease degradation, again a property thought largely restricted to PrP^Sc^, has been reported for some forms of α-synuclein [141]. Because of such parallels with prion diseases, a new terminology was introduced for the group of pathogenic proteins that underlie these neurodegenerative disorders and includes prion-like, prionoid, quasi-prion and propagon [7,142,143]. Whilst there remains some debate surrounding this terminology, the rationale was to denote a group of proteins with the propensity to misfold and aggregate homotypic molecules, as described in prion diseases, but in the absence of any demonstrable infectivity [144]. However, such commonalities have inevitably raised the question over the potential human transmissibility of pathogenic protein aggregates other than PrP^Sc^, which would have significant public health implications.

### 3.1. Experimental Animal Models of Pathogenic Protein Transmission

Over the last decade, a wealth of experimental data has been published addressing the potential transmissibility of neurological conditions and the misfolded protein aggregates that underlie them. Such studies have largely been based on experimental approaches used in the demonstration of the transmissible nature of prion diseases. A significant number of these investigations focus on Aβ, tau, and α-synuclein, the proteins that underlie some of the most commonly occurring neurodegenerative diseases.

Reminiscent of prion diseases, the earliest studies involved the direct inoculation of diseased brain material from patients with different neurological conditions into non-human primates. Mirroring their seminal work on kuru and CJD, Gajdusek and Gibbs investigated the potential transmissibility of AD with the direct inoculation of brain tissue from 52 patients with AD (familial and sporadic AD cases) into non-human primates. Whilst no evidence of a clinical disease was reported in any animal, neuropathological analysis of a proportion of animals inoculated with brain isolates from two familial AD (fAD) patients did show a pathology that was indistinguishable from that of prion disease [145]. However, as these results were not replicated in subsequent experiments, it has been hypothesised that the presence of prion pathology in these animals was most likely a result of unrecognised and co-existing prion disease in the two fAD patients and unrelated to AD pathology. In a later publication summarising data from the National Institutes of Health’s (NIH) 30-year-long series of experimental transmission of human prion disease in non-human primates, Gajdusek and colleagues included data from the inoculation of brain isolates from over 600 patients who were diagnosed with a wide variety of neurological disorders including MND, HD, PD with dementia, and Picks disease [146]. In this large-scale study, no conclusive evidence of disease transmission was reported from any of the non-prion related neurological conditions, even after post-inoculation intervals of more than nine years. These two studies provide supporting evidence that prion diseases are unique amongst neurodegenerative conditions in their ability to recapitulate a clinical disease in non-human primates. However, a caveat to both studies, remains a lack of in-depth neuropathological investigations and as such have not addressed the question of whether the associated protein pathology may be transmissible in non-human primates, as in prion disease. In two later studies, clinical signs of disease were again absent but Aβ aggregation was observed in the brains of non-human primates at post-mortem following the intracerebral injection of Aβ containing brain extracts from AD cases [147,148]. The Aβ pathology was present in the brain parenchyma (Aβ plaques) and in cerebral blood vessels as cerebral amyloid angiopathy (CAA). Age-matched control animals did not show these neuropathological changes. While Aβ pathology was observed in the majority of inoculated animals, tau aggregates, also a major protein associated with AD, was not reported in either study [147,148]. In a more recent study, intracerebral inoculation of AD brain isolates in a different non-human primate model demonstrated that both Aβ and tau aggregates can be induced in the brain. Furthermore, longitudinal cognitive assessments, electroencephalography (EEG), and morphological magenetic resonance imaging (MRI) performed at a series of time points post-inoculation showed neuronal loss, progressive atrophy, and alteration of neuronal activity in the animals, as well as evidence of cognitive impairment [149]. In addition to Aβ and tau from AD tissues, α-synuclein aggregation was induced in non-human primates by the intracerebral injection of Lewy body-rich extracts from the brains of PD patients. The resulting α-synuclein pathology extended beyond the site of inoculation and, consistent with PD pathology, neurodegeneration and α-synuclein pathology in the recipient brain was most marked in the dopaminergic neurons of the substantia nigra [150,151].

Like prion diseases, the development of transgenic mice expressing different transgenes has transformed the ability to investigate the potential for the transmission of other neurological conditions and the specific protein aggregates that underlie them [152]. Many of the observations from these transgenic mouse models have strengthened the evidence for fundamental similarities between aspects of the pathology of prion diseases with the pathology of other neurodegenerative conditions. The production of mice expressing the amyloid precursor protein (APP) gene has provided a useful model in which to examine the intracerebral seeding of Aβ pathology in mice inoculated with exogenous, misfolded Aβ from AD patients. Such Aβ seeding was first reported in the early 2000s by Lary Walker and colleagues, who described Aβ aggregation in the brain parenchyma of APP-transgenic mice intracerebrally inoculated with Aβ-rich extracts from patients with AD disease [153,154]. The Aβ pathology extended beyond the site of injection and in some mice, contralateral to the site of injection, indicating the cell-to-cell spread of the aggregated protein through defined neuroanatomical pathways [153,154]. Numerous studies using multiple transgenic lines have since reported Aβ seeding in the brain following intracerebral injection of AD brain isolates. Of particular significance was the observation that Aβ aggregation was induced in transgenic mice that do not spontaneously develop Aβ pathology in their lifetime [155]. Collective data from these studies have demonstrated that (i) Aβ can aggregate in the brain parenchyma (Aβ plaques) and within the cerebral blood vessels, (ii) the accumulation of Aβ in the brain is time and concentration-dependent [156], (iii) Aβ aggregation in the brain occurs even using sub-attomolar concentrations of Aβ from AD patients [157], (iv) Aβ aggregation may be induced from Aβ-rich brain samples from patient who had AD, patients showing mild cognitive impairment and from patients who had no evidence of cognitive decline but who had AD pathology in their brain [158] and, (v) the seeding of Aβ in the brain can be induced by the introduction of exogenous Aβ via the intravenous route but with a slower time-course than that of intracerebral inoculations [159]. Aβ aggregates have also been induced following intraperitoneal inoculation of transgenic mice, but these studies did not use Aβ seeds of human origin [160,161]. Whilst many of these properties are reminiscent of that described in prion diseases, the question of whether the strain phenomenon is applicable to other misfolded proteins is an important and current focus of research. APP transgenic mouse models have added to the evidence for different conformations of Aβ following the demonstration of distinct transmission properties (incubation period and patterns of neuropathology) in these mice following intracerebral inoculation with brain material from fAD and sporadic AD. Crucially, these strain-like properties were stable on serial passage [162].

As with the generation of APP mice in the study of Aβ transmission, several transgenic mouse lines have been generated to model the seeding of tau pathology. Numerous studies have demonstrated that the intracerebral inoculation of brain tissue from individuals with AD and different tauopathies, examples of which are; progressive supranuclear palsy (PSP), corticobasal degeneration (CBD), and argyrophilic grain disease (AGD) (Figure 1), can induce the formation of tau aggregates in both tau-transgenic mice and in wild-type mice [163,164]. The ability to induce tau seeding in wild-type mice is in contrast to Aβ aggregates and provides a valuable model in which to study the seeding of tau pathology in mice that do not spontaneously develop tau pathology in their lifetime. Many of the transmission properties of tau are reminiscent of that demonstrated by Aβ and PrP^Sc^ in transgenic mice. Intracerebral inoculation of tau-containing brain extracts showed tau pathology spreads in the brain via the cell-to-cell spread and propagation from the site of injection to the surrounding brain regions. Frequently, the morphology of the newly formed tau aggregates in the brain emulates the pathology found in the human tauopathy source. This is best observed in transgenic mice inoculated with brain isolates from AD cases, where much of the pathology targets the hippocampus. Reminiscent of prion disease, inoculation of brain isolates from different tauopathies induced tau pathology in mice that differed in morphology, regional distribution, cell-type specificity, and pattern and rate of spread. Each of these properties indicate that tau, like PrP^Sc^ and Aβ, can adopt multiple molecular conformations giving rise to different prion strains. [165,166,167]. Again, these different tau conformations are stable on subsequent passage. Like Aβ, tau aggregates are also seeded by the intraperitoneal inoculation of exogenous tau, but this has not been demonstrated using tau aggregates from human brain isolates [168]. While the seeding potential of PrP^Sc^ and Aβ has been shown to favour smaller soluble oligomers, the seeding potential of tau aggregates is thought to favour the larger insoluble fragments.

Other misfolded protein aggregates investigated by the generation of transgenic mouse models includes α-synuclein, which underlies PD, dementia with Lewy bodies (LBs), and multiple system atrophy (MSA). Like Aβ and tau in AD, α-synuclein pathology in PD follows a distinct and consistent pattern of progression in the diseased brain. This pattern of progression is indicative of a similar cell-to-cell spread of α-synuclein. Compelling evidence for this came from the observation of α-synuclein pathology in healthy nigral neuron cells, 14 years after they were grafted into the striatum of a PD patient [169]. Following on, α-synuclein-rich isolates extracted from the brains of PD patients were sufficient to induce the seeding of pathological α-synuclein in wild-type mice [150,170]. Similarly, α-synuclein derived from the brains of patients with MSA were also effective in at seeding α-synuclein pathology and in some instances, were sufficient to produce a neurological disease [171]. Whilst limited studies investigating the presence of α-synuclein strains are available in mouse models, differences in the incubation period and pattern of neuropathology in transgenic mice inoculated with MSA isolates was indicative of separate α-synuclein strains [171]. This was supported by the failure to induce α-synuclein pathology in the same mice inoculated with α-synuclein-rich extracts from PD.

### 3.2. Human Transmission of Pathogenic Proteins

The inadvertent human-to-human transmission of prions associated with a number of medical interventions raised the possibility that other misfolded proteins may be capable of transmitting via similar iatrogenic sources of infection (Figure 2). Treatment with contaminated human pituitary-derived growth hormone is one of the most commonly associated causes of iCJD. Reports of Aβ, tau, and α-synuclein aggregates in the pituitary gland of some elderly patients and in some patients with neurodegenerative conditions [172,173,174], prompted a retrospective review of the National Hormone and Pituitary Program (NHPP) cohort database and the medical literature to establish an association between neurodegenerative conditions and a history of hGH treatment [174]. While no association was reported, the study was limited by a lack of neuropathological evidence on the presence of protein aggregates in the brain of the hGH recipients. This was subsequently addressed in four independent reports examining a total of 80 iCJD-hGH cases originating from the UK, France, and USA [175,176,177,178]. Of the 80 cases, 73 had sufficient post-mortem fixed tissue samples for full neuropathological analysis, of which 26 (36%) reported substantial Aβ pathology consistent with AD in the brain parenchyma and/or in cerebral blood vessels. No clinical manifestations of AD were identified in any of the hGH recipients. The co-occurrence of Aβ and PrP^Sc^ aggregates has been reported previously in human prion diseases, particularly in genetic prion diseases that are associated with PrP^Sc^ amyloid plaque formation [179,180,181]. As all 73 cases had clinical iCJD, the possibility that the Aβ aggregates may have resulted from cross-seeding with the co-existing PrP^Sc^ was raised. However, in one of the largest studies, Ritchie and colleagues reported Aβ pathology in the brain of 5 out of 12 hGH recipients who died of causes unrelated to CJD [176]. This data, combined with the lack of evidence of any associated genetic risk factor in the development of Aβ pathology and the relatively young age (<55 years) of the hGH-iCJD patients, were highly indicative that the Aβ in these patients was linked to exogenous Aβ aggregates present in the hGH. This was supported following the detection of substantial levels of Aβ and tau contaminants in some of the archived hGH batches analysed from the UK and France [177,182]. Furthermore, experimental transmission of samples of UK hGH extracts in transgenic mice expressing APP showed that these extracts had sufficient levels of Aβ to induce the seeding of Aβ in the brain [182]. While tau contaminates were also identified in the hGH batches, little evidence of tau pathology has been identified in the hGH recipients investigated, perhaps indicative of a different biological mechanism in the seeding of tau pathology.

A history of cadaveric Dura Mater grafting is another medical intervention commonly associated with iCJD cases worldwide (Figure 2) [118]. Co-existing pathology in DM-iCJD patients was first reported in 2006 with the description of Alzheimer-type senile plaques and CAA in the brain of a 28-year-old iCJD patient who had received a dura mater graft in childhood [183]. The Aβ accumulation was initially reported as an incidental finding, perhaps related to the early trauma in the brain. It was a further 10 years until the possibility was raised that the Aβ pathology in this patient may have seeded from exogenous Aβ aggregates present in the grafted dura mater [184]. Since this report, substantial Aβ pathology has been described in the brain parenchyma and cerebral blood vessels in a further 28 out of 38 DM-iCJD patients investigated with sufficient post-mortem tissue, after post dura mater graft transplant intervals of over 20 years. [178,184,185,186,187]. Regardless of the relatively young age of the patients and the high prevalence of Aβ pathology, the presence of co-existing PrP^Sc^ raised the possibility of cross-seeding in the brain. However, the demonstration of Aβ deposits in the grafted dura mater tissue in some of these cases, and the significant association with subpial Aβ deposition and meningeal amyloid angiopathy, support a causal relationship between dura mater grafting and Aβ accumulation. [184]. This is further supported by three recent independent publications reporting intracerebral haemorrhage associated with sporadic CAA in five young individuals (<48 years) who received a cadaveric dura mater graft in childhood, in the absence of any evidence of CJD [188,189,190].

Whilst only a small number of iCJD cases worldwide have been associated with contaminated neurosurgical instruments, the potential for this to be a mechanism of inducing Aβ aggregation in the brain was raised following the observation of Aβ accumulation in the brains of APP transgenic mice intracerebrally challenged with Aβ-contaminated steel wires [191]. Following reports on the cadaveric dura mater graft recipients, diagnosis of intracerebral haemorrhage associated with CAA in young individuals was adopted in a recent investigation looking at other neurosurgical interventions as a potential mechanism in the transmission of Aβ pathology. A retrospective review of the medical literature and neuropathology archive at the National Hospital for Neurology and Neurosurgery (NHNN) identified seven individuals under the age of 42 years and one 57-year-old patient who presented with intracerebral haemorrhage associated with CAA, all of which had undergone neurosurgical procedures in childhood [192]. With no associated genetic risk in developing early onset CAA, the possibility that the Aβ pathology was induced by Aβ aggregates present on the neurosurgical instruments has been proposed. This has been supported by two subsequent case reports of CAA-related intracerebral haemorrhage in three patients, under 30 years of age with a history of neurosurgery in childhood [193,194].

There is no definitive evidence to suggest that sCJD is transmissible via blood transfusion [125,126]. However, secondary transfusion-transmission of vCJD infectivity has been described in four individuals [124,127,128]. In the context of transfusion-associated risks for other neurodegenerative conditions, there is no current evidence that blood transfusion is a risk factor in the development of disease. However, with increased levels of plasma Aβ reported in blood from elderly donors and a lack of experimental studies, the possibility of transfusion transmission of AD or AD pathology, after an extended incubation period cannot be discounted and supports further investigation [195,196].

## 4. Concluding Remarks and Future Perspectives

Prion diseases remain unique among neurodegenerative conditions, with the potential to transmit disease from person-to-person as rare events after a prolonged incubation period. Lessons learned from instances of acquired prion diseases have resulted in the implementation of a range of safety measures in order to prevent future iatrogenic transmission of CJD, further secondary transmission of vCJD, and the potential zoonotic spread from other animal prion diseases. However, with prevalence studies suggesting that a significant number of the UK population may be harbouring asymptomatic vCJD infectivity, and with the appearance of vCJD in a patient heterozygote for methionine and valine at PRNP codon 129, prion diseases remain a considerable public health concern. Of additional concern is mounting evidence that other misfolded proteins, specifically Aβ, can transmit from person-to-person in a prion-like mechanism of propagation and spread through similar routes of exposure to those described in iCJD. In contrast to prion diseases, the full clinical and neuropathological phenotype of AD has not been reproduced in these individuals; in particular, there is a notable absence of neurofibrillary tangles and progressive cognitive decline. While this may be reassuring, the ability to induce Aβ aggregation in the brain through medical interventions may have wider implications for public health. In particular, current reports of substantial Aβ seeding in the cerebral blood vessels in patients, decades after neurosurgical interventions, suggest there may be future vascular complications associated with iatrogenic-CAA, including intracerebral haemorrhage, perivascular inflammation, and cognitive impairment [197]. Of additional consideration is the potential transmission of prion or prion-like pathology through the possible contamination of newly developed advanced cell therapies (ACT). These concerns and implications were recently addressed in a detailed review by De Sousa et al. [198]. While experimental evidence from animal mouse models has demonstrated that tau and α-synuclein, like Aβ, can be seeded from the brains of patients with a range of tauopathies and synucleinopathies, there remains no evidence for the human transmission of tau or α-synuclein pathology between individuals. As evidence for the transmission of pathogenic proteins are based on small cohorts of patients or on single case reports. The implementation of large-scale, systematic studies are necessary in order to adequately assess the potential risks associated with the potential transmission and propagation of misfolded protein in humans.

## Data Availability

No new data were created or analyzed in this study. Data sharing is not applicable to this article.
